# A Cross-Sectional Study of the Relationships between Work-Related Affective Feelings Expressed by Workers in Turkey

**DOI:** 10.3390/ijerph17249470

**Published:** 2020-12-17

**Authors:** Erman Çakıt, Waldemar Karwowski, Tadeusz Marek, Magdalena Jaworek, Grzegorz Wrobel

**Affiliations:** 1 Department of Industrial Engineering, Gazi University, Ankara 06570, Turkey; 2 Department of Industrial Engineering and Management Systems, University of Central Florida, Pegasus Dr., Orlando, FL 12800, USA; wkar@ucf.edu; 3 Institute of Applied Psychology, Jagiellonian University, 30-252 Krakow, Poland; marek@uj.edu.pl; 4 Institute of Economics, Finance, and Management, Jagiellonian University, 30-252 Krakow, Poland; magdalena.jaworek@uj.edu.pl; 5 Faculty of Management, University of Information Technology and Management, 35-225 Rzeszów, Poland; gwrobel@wsiz.rzeszow.pl

**Keywords:** work-related affective feelings, feelings at work, PLS-SEM, modeling, Turkey

## Abstract

Understanding employees’ feelings at work plays a significant role in developing practical and effective organizational and human resource management policies and practices. Furthermore, work-related emotions may have a considerable effect on workers’ health and wellbeing and affect work effectiveness and work performance. The objectives of the current study were to investigate the relationships among four work-related (WOR) affective feelings (WORAF) and to validate the WORAF questionnaire in a Turkish sample. A survey was performed including four constructs: (1) WOR feelings of happiness, (2) WOR feelings of anxiety, (3) WOR feelings of anger, and (4) WOR feelings of dejection. A total of 322 workers from various companies in Turkey completed a paper-based survey. A research model was developed, and its main components were estimated with partial least squares structural equation modeling (PLS-SEM). The results revealed that dejection and anger at work play a critical role in experienced anxiety in occupational settings. Similarly, dejection, anger, and anxiety at work play a crucial role in perceived happiness at work.

## 1. Introduction 

Understanding the roles of employees’ feelings is critical to effective human resource management in contemporary organizations, because most individuals in the population spend more than 50% of their lives at work [1]. A wide variety of affective responses is correlated with employment and can involve general positive feelings (pleasure, happiness) or negative (displeasure, frustration) and other feelings, including anger, frustration, joy, and excitement. This study focuses on investigating feelings of happiness, anxiety, anger, and dejection. These feelings are recognized as the most frequently expressed emotions and are considered fundamental emotions [2,3,4,5]

Contemporary research has shown that descriptions such as “positive” or “negative” emotions can be replaced by emotion-related terms when they apply to real-life circumstances or situations (e.g., workplace situations) [6]. Lindebaum and Jordan [7] have observed a propensity to investigate symmetrical relations between distinct emotions that are viewed positively and negatively. Alternatively, they suggest that asymmetries of feelings should be studied in the workplace. From the viewpoint of behavioral psychology and organizational behavior, emotions can be associated with managerial actions in several aspects. For example, Frost [8] has indicated that dissatisfied workers appear to become detached from their jobs. In addition, if individuals do not recognize the relational aspects of corporate behavior, they are unlikely to be aware of future counterproductive acts, such as unjust business policies or hostile managers.

Although emotions or feelings have been studied across numerous epistemology systems, including neuroscience, social psychology, cognitive science, and philosophy, the mechanisms of human affective perception remain poorly understood. Little research has been conducted on the specific roles of feelings, particularly the critical connections of positive and negative emotions in organizations. A substantial research gap exists concerning the relationship between positive and negative feelings at work. Recently, Jaworek et al. [9] developed and validated a new instrument to assess four work-related (WR) affective feelings (WORAF). The current study’s objectives were to validate the WORAF questionnaire in a Turkish sample and to investigate the relationships among four WORAFs: happiness, anxiety, anger, and dejection. In addition, the social and psychological factors that influence workers’ decisions and might better forecast employee expectations, attitudes, and behaviors were considered.

## 2. Background

Consensus is currently lacking as to what constitutes a feeling, how feelings can be evaluated, or how the effects can be applied in workplaces through the analysis of feelings [10]. Research has convincingly shown that feelings play important roles in working conditions, organizational behavior, and leadership [11,12]. Feelings are known as individual and subjective emotions that may trigger various behavioral events or reactions. Therefore, feelings may be interpreted as distinct, relating to emotions that are commonly felt, such as anger or pleasure, which are assumed to correspond to particular facial expressions [13]. 

Social and sociocultural theories posit that feeling as a personal phenomenon clearly lacks the importance of complex social and cultural backgrounds and the internal, communicative role of emotions [14]. Feelings are also not only processes in the mind but also entities, according to socially orientated viewpoints, that shape and organize social interactions and their effects [15]. The fundamental concept is that the expression of feelings relies on experienced, socially and culturally defined laws [16]. Feelings may also be interpreted as social structures that have a contextual label relating to definitions created by culture and ideas underlying tradition and daily experience [17]. Feelings are also seen as dynamic mechanisms that form social activities, experiences, and their effects—for example, in the context of the workplace.

Researchers have examined feelings from psychological and sociological perspectives in the field of organization and organizational research. In this field, the principles of emotional intelligence and feelings at work have been addressed. Emotional intelligence refers to the degree to which one can manage one’s emotions and correctly direct one’s own thoughts and acts [18,19]. This process requires an ability to consider and identify the feelings of others and to use this knowledge to direct decision-making and behavior [18]. Feelings at work [20] encompass the study of (i) how displays of feeling, as part of individuals’ work roles, are used to influence others to accomplish organizational goals, such as increasing customer satisfaction [21], or (ii) how organizationally acceptable feelings can be generated [22]. 

In general, feeling at work requires workers to control or maintain their emotional behavior, in terms of the substance, frequency, severity, and length of emotions [23]. Therefore, this term refers to cases in which workers are expected to express feelings that differ from what they really experience [24]. In general, one might argue that philosophical debate and scientific research (particularly in organizational and workplace studies) have conceptualized feelings in several ways, whereas feelings have been traditionally considered to be socially formed and regulated.

Various definitions have been proposed to explain the concept of happiness at work. For example, Fisher [25] has stated that workplace happiness reflects positive attitudes or pleasant experiences at work. Happiness is attributed to peoples’ willingness to perceive circumstances as less unpleasant and consider and manage their emotions. The ability to cope with negative feelings can, among other aspects, have a positive effect on morale and promote mental health; conversely, depressive symptoms threaten wellness. Furthermore, the impact of negative feelings has been explored by Sargeant et al. [26], who have suggested that receiving negative reviews at work often evokes negative emotions. Several researchers have confirmed the close relationships among anger, anxiety, and dejection. Lazarus and Cohen Charash [27], for instance, have suggested that anger and anxiety are “strictly interdependent”. Some researchers note that dejection and anger can also be encountered together [28,29]. The energy of anger counteracts the slowing effect of dejection, and dejection, in turn, moderates the anger’s intensity [28].

**Figure 1:**
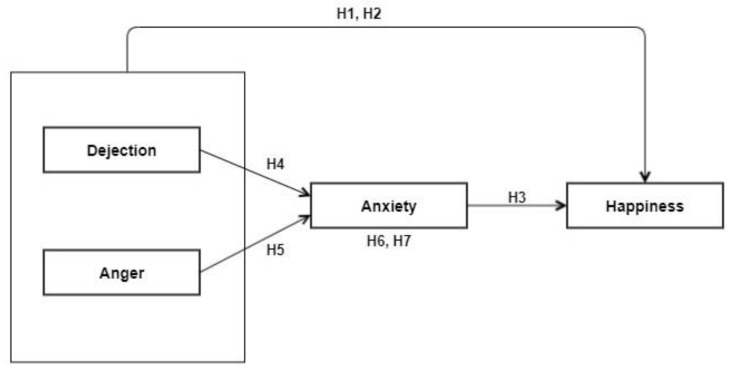
The hypothesized conceptual model.

In view of the above discussion, the relationships among happiness, dejection, anger, and anxiety were explored on the basis of the following seven hypotheses (Figure 1):

**Hypotheses 1** **(H1).** *There exists a significant negative relationship between dejection and happiness.*

**Hypotheses 2** **(H2).** *There exists a significant negative relationship between anger and happiness.*

**Hypotheses 3** **(H3).** *There exists a significant negative relationship between anxiety and happiness.*

**Hypotheses 4** **(H4).** *There exists a significant positive relationship between dejection and anxiety.*

**Hypotheses 5** **(H5).** *There exists a significant positive relationship between anger and anxiety.*

**Hypotheses 6** **(H6).** *Anxiety mediates the relationship between dejection and happiness.*

**Hypotheses 7** **(H7).** *Anxiety mediates the relationship between anger and happiness.*

## 3. Methods and Procedure 

### 3.1. Study Design

In this cross-sectional study, Turkish employees in different occupations participated in a survey on emotions in the workplace. A cover letter was sent with an invitation to participate in the survey, and an informed consent form was required for participation. The Institutional Review Board of Gazi University, Ankara, Turkey, accepted the survey questionnaire and the experimental protocol (#91610558-604.01.02). The original questionnaire was published in a prior study and validated in a large group of Polish employees in different occupations [9]. This questionnaire was translated into Turkish, and a preliminary test of the questionnaire in a small group of Turkish workers was conducted. This confirmation phase ensured that the participants answered all the claims in the translated edition of the survey without any problems.

### 3.2. Study Variables

The questionnaire was divided into two sections. The first part asked respondents to provide demographic information, such as gender, age, education, occupation, working hours, and work experience. The second part included questions with answers calculated on a four-point Likert scale ranging from (1) (almost) never, to (4) (almost) always. To address missed and unanswered details, the surveys of participants who did not complete all the survey statements were omitted and not included in the final dataset. With the information described above in the questionnaire, the initial set of variables used for model development is described in Appendix A.

### 3.3. Participants

A total of 575 workers were invited to participate in the research. Of these, 322 respondents (173 (53.7%) male workers and 149 (46.3%) female workers) provided valid surveys, thus resulting in a response rate of 56%. Participants worked in a variety of industries and organizations in Turkey. In the survey, the main occupational groups were as follows: 146 engineers (45.3%), 70 (21.7%) managers, 64 (19.9%) office workers, and 42 (13.1%) people reporting associations with other professions. The age ranged from 19 to 59 years. The age distribution included 56 (17.4%) respondents under 25, 98 (30.4%) between 25 and 30, 136 (42.3%) between 31 and 40, and 32 (9.9%) over 40 years of age. Regarding work experience, 127 (39.4%) of the respondents had worked less than 5 years, 92 (28.6%) had worked between 5 and 10 years, 81 (25.2%) had worked between 11 and 20 years, and 22 (6.8%) had worked more than 20 years (Table 1). With regard to education level, 48 (14.9%) of the respondents had graduated from high school, 218 (67.7%) were college graduates, and 56 (17.4%) had earned a Master’s degree or Ph.D.

### 3.4. Statistical Analysis

Descriptive statistics and frequency analysis of demographic information were performed in IBM SPSS (v.25), and other statistical analyses were conducted in SmartPLS (v.3.3.2) software [30,31]. Multicollinearity analysis, confirmatory factor analysis (CFA), reliability and convergent validity, discriminant validity, path coefficients, hypothesis testing, and PLS-SEM were used to investigate the relationships among model factors.

## 4. Results

### 4.1. Multicollinearity Analysis

We calculated the means and standard deviations for all research variables as the first step of model testing. A correlation analysis was also performed to determine the relationship between any two variables used in model development (Table 2). All model variables had significant relationships at the *p* ≤ 0.01 level. There was a positive correlation between anxiety and dejection and also between anxiety and anger. In addition, there was a negative relationship between happiness and anxiety and also between happiness and dejection. Multicollinearity was described as a high correlation among two or more constructs [32] and was verified by an indicator of variance inflation factors (VIF). To calculate the VIF values for all exogenous variables in the data group, we performed analysis in SmartPLS (v.3.3.2). As recommended by Hair et al. [31], all VIFs were less than 5.0 (<5.0) and thus were deemed acceptable measures. In general, if the VIF coefficient is greater than 5.0, a problem of multicollinearity might exist. None of the VIF coefficient values in the model results exceeded the threshold value of 5.0, thus indicating that no collinearity problems were found in the model results (Table 3).

### 4.2. Confirmatory Factor Analysis

**Figure 2:**
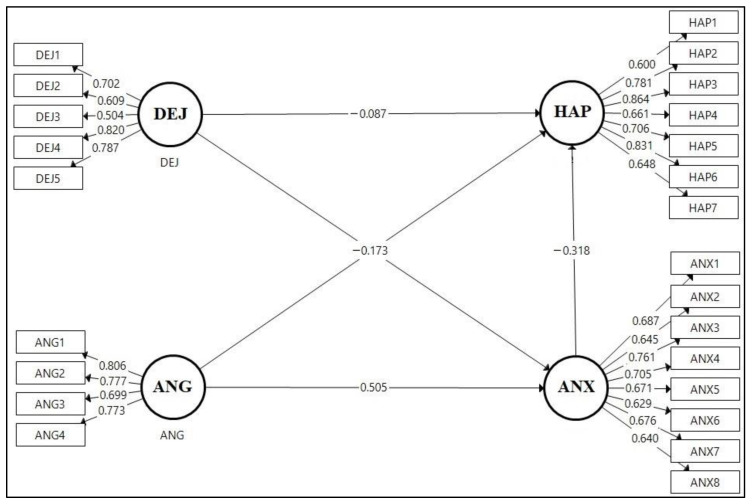
An initial structural model with outer loading.

**Figure 3:**
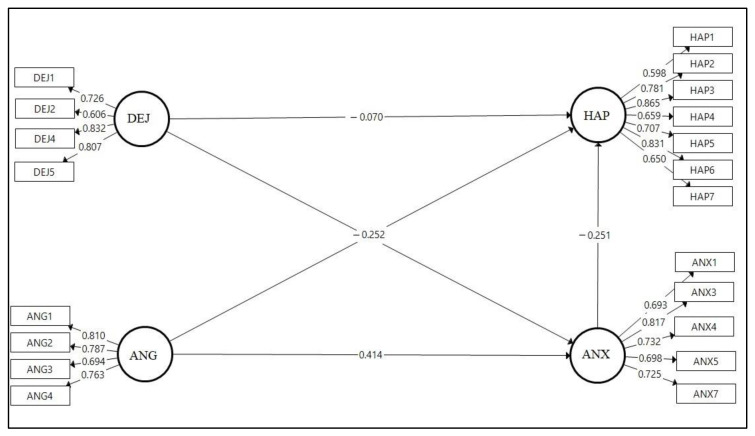
A final structural model with outer loading.

The validation of the measurement model was performed through assessment of relationships between the constructs and their respective measurement elements with confirmatory factor analysis (CFA). The initial model consisted of several individual items with low indicator loadings (Figure 2). An initial CFA was conducted, and according to the results, we extracted several items and then reran the model. In total, we deleted three items from ANX (ANX2, ANX6, and ANX8) and one item from DEJ (DEJ3), on the basis of low indicator loadings, to increase the construct’s reliability and validity. The CFA was performed again with the remainder of the measurement items (Figure 3).

### 4.3. Reliability and Convergent Validity

To determine the reliability, validity, and path coefficients of our model, we conducted SmartPLS (version 3.3.2, Bönningstedt, Germany) analysis. The Cronbach alpha and composite reliability were used for reliability analysis, as suggested by Fornell and Larcker’s [33] and Cronbach’s [34] criteria. To test the validity of the model, we applied convergent and discriminant validity as part of the construct validity. The convergent validity determines how precisely the latent model represents the scale items [35]. Fornell and Larcker [33] have used their average variance extracted (AVE) criterion to establish convergent validity. The AVE is the component of the knowledge that each structure explains with respect to their variable classes or how often, on average, the variables are positively associated with their specific constructs [36]. In this respect, as stated by Hair et al. [37], an AVE > 0.50 indicates that each reflective construct describes more than 50 percent of the variance of its objects. The final structural model was found to satisfy all relevant reliability and validity requirements, as follows: Cronbach’s alpha > 0.7; rho_A > 0.7; composite reliability (CR) > 0.8; and average variance extracted (AVE) > 0.5. Table 4 shows the detailed findings.

### 4.4. Discriminant Validity

Discriminant validity, as demonstrated in Table 5 and Table 6, reveals that “each construct represents its own dimension and the model constructs are clearly distinct, with each construct sharing more variance with its associated items than with any other construct” [37]. In this respect, the square root of the AVE of each construct (diagonal entries in Table 5) was greater than its absolute similarity with any other construct (nondiagonal entries in Table 5), thus suggesting that the calculation model followed the discriminating validity requirements that Fornell and Larcker [33] suggested. Table 5 and Table 6 demonstrate that each building reflects its own axis, and the model constructions are distinctly distinct: “each building shares more variation with its related objects than any other construction”. In addition, the measurement model met the HTMT.90 discriminant validity criteria established by Henseler et al. [38] and the heterotrait–monotrait (HTMT) ratio of correlations being lower than 0.90 (Table 6).

## 5. Hypothesis Testing Results and Discussion

**Figure 4:**
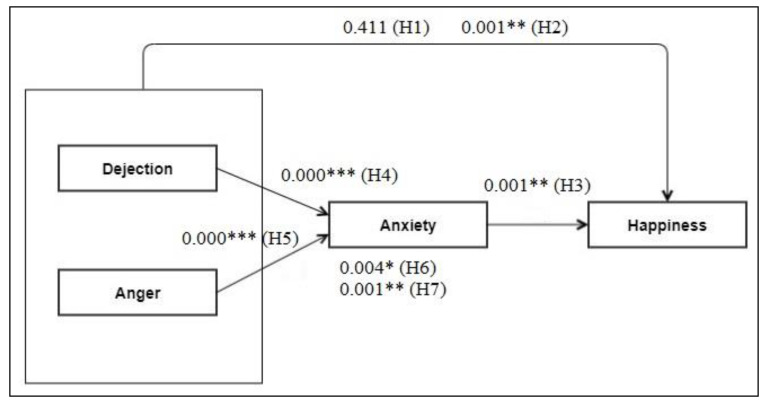
Final work-related affective feelings (WORAF) model (*p*-values). Note: * *p* < 0.05. ** *p* < 0.01. *** *p* < 0.001.

We conducted path analysis for all latent predictors to evaluate the correlations between each latent variable given the examined research hypotheses. Moreover, we conducted a bootstrapping test (5000 subsamples were generated) to measure the validity of path coefficients by using PLS-SEM and to calculate t-test values. After the validation of the measurement model, path coefficients (β), t-values (t), and *p*-values (*p*) were obtained to determine whether the hypotheses were appropriate. Table 4 displays the estimated path coefficients and t-values between the latent variables. Most of the hypotheses were confirmed by the findings of the study, except for H1. The analyses above yielded the findings below (Table 7 and Figure 4):

- Dejection at work was not statistically significantly associated with happiness at work (β = −0.065; *p*-value > 0.05); thus, H1 was rejected;
- Anger at work negatively influenced happiness at work (β = −0.252; *p*-value < 0.05), thus supporting H2;
- Anxiety at work had a significant negative relationship with happiness at work (β = −0.255; *p*-value < 0.05), thus supporting H3;
- Dejection at work had a significant positive relationship with anxiety at work (β = 0.435; *p*-value < 0.05), thus supporting H4;
- Anger at work positively influenced anxiety at work (β = 0.416; *p*-value < 0.05), thus supporting H5;
- Anxiety at work mediated the relationship between dejection and happiness (β = −0.111; *p*-value < 0.05), thus supporting H6;
- Anxiety at work mediated the relationship between anger and happiness (β = −0.106; *p*-value < 0.05), thus supporting H7.

**Figure 5:**
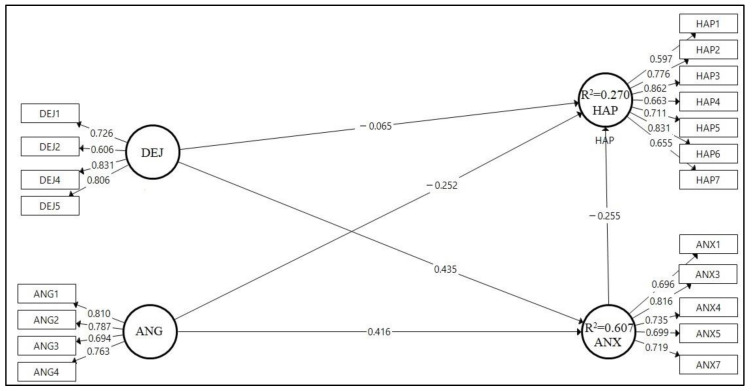
Final structural model with standardized path coefficients.

We also calculated the R^2^ of the model to assess the amount of change in the dependent constructs caused by the independent variables. We also hypothesized that the set of variables included in the experiment plays a key role in feelings at work. From the findings of the research and bootstrapping results, we inferred that dejection and anger at work play a key role in anxiety at work. Changes in dejection and anger at work were found to affect anxiety, with R^2^ = 0.607. That is, anxiety was affected by dejection and anger at work, with a contribution of 60.7%. Likewise, dejection, anger, and anxiety at work were found to play a crucial role in happiness, with R^2^ = 0.270 (Table 7 and Figure 5).

The findings of the study demonstrate the patterns illustrated in Figure 5. The final model can be summarized as follows:

- As anger at work increases, happiness at work decreases.
- As anxiety at work increases, happiness at work decreases.
- As dejection at work increases, so does anxiety at work.
- As anger at work increases, so does anxiety at work.
- Anxiety at work mediates the relationship between dejection and happiness at work.
- Anxiety at work mediates the relationship between anger and happiness at work.

The theoretical findings and outcomes of our study are supported by empirical experiments performed with a dimensional approach, thus suggesting that three of the observed work-based feelings are closely linked [39,40,41].

Overall, the current results are encouraging and enable further studies to be performed by using the measuring instrument applied herein. The WORAF included only the affective states selected in this study. Further studies examining different forms of work-related emotions, including remorse, embarrassment, envy, optimism, ambition, sympathy, and even love, should also be performed, as proposed by other researchers [27]. Because all participants were Turkish, the findings cannot be generalized to people of other nationalities, particularly because the constructs examined can be affected by culture and society [42]. Therefore, additional experiments examining working classes other than Turkish workers on the WORAF scale are also highly recommended.

The findings of several studies [43,44,45] indicate that the effective management of employees’ feelings leads to numerous positive results, including a lower risk of depression [46], greater control over the expression of violence [47], a more developed sense of morality [48], and improved psychological development [49], among others. Oktug [50] demonstrated that managing emotions in the workplace could reduce employees’ stress levels and pointed out that emotion management is one of the dimensions of emotional intelligence. Rosete and Ciarrochi [51] reported that higher emotional intelligence was associated with leadership effectiveness. Furthermore, a recent study by [52] revealed that project managers who feign emotional expressions as a strategy to regulate emotions in the workplace experienced higher levels of stress and encountered job burnout.

An essential part of emotional regulation requires understanding the relationships among four work-related affective feelings investigated in the current study. Organizations can use such knowledge to manage emotions at work and mitigate their potential adverse effects by developing effective strategies for success, including higher leadership effectiveness, prevention of employee burnout, and increased job performance.

## 6. Conclusions

This research’s primary objectives were to validate the WORAF questionnaire in a sample of employees in Turkey and assess the relationships among four work-related emotions: happiness, anxiety, anger, and dejection. A cross-sectional quantitative methodology with a correlational design was used for this purpose. The study results revealed that dejection and anger at work play a critical role in expressing anxiety at work. Changes in dejection and anger at work were found to affect anxiety. Similarly, dejection, anger, and anxiety at work were found to be related to the level of happiness. It is concluded that the WORAF scale can improve the understanding of individuals’ emotional functioning in work environments.

It should be noted that all the study participants were Turkish, so that these findings cannot be generalized to include other nationalities. Therefore, additional WORAF-scale-based investigations using working communities other than Turkish workers are strongly recommended. Further studies on affective feelings at work should be based on both situational and permanent dimensions and should aim to incorporate interventions within and between individuals. The WORAF scale could also be used to develop similar instruments to assess other affective states concerning particular conditions at work. Finally, gender differences in emotion management in the workplace should also be examined.

## Figures and Tables

**Table 1:** Profile of respondents.

Demographic Variable	All (*n* = 322)	
	Frequency	(%)
Gender		
1. Male	173	53.7
2. Female	149	46.3
Age		
1. Less than 25	56	17.4
2. 25–30	98	30.4
3. 31–40	136	42.3
4. Older than 40	32	9.9
Work experience		
1. Less than 5 years	127	39.4
2. 5–10 years	92	28.6
3. 11–20 years	81	25.2
4. More than 20 years	22	6.8

**Table 2:** Means, standard deviations, and correlations.

Variable	Mean	S.D.	ANX	HAP	DEJ	ANG
ANX	1.68	0.52	-	-	-	-
HAP	2.93	0.69	−0.51	-	-	-
DEJ	1.53	0.53	0.71	−0.42	-	-
ANG	1.73	0.57	0.75	−0.47	0.66	-

Notes: Correlations are significant at the *p* ≤ 0.01 level. Abbreviations: Anxiety (ANX); happiness (HAP); dejection (DEJ); anger (ANG).

**Table 3:** Variance inflation factors (VIF) values for all exogenous variables.

	VIF		VIF		VIF
ANG1	1.620	HAP1	1.296	ANX1	1.662
ANG2	1.592	HAP2	2.060	ANX2	1.409
ANG3	1.361	HAP3	2.903	ANX3	1.879
ANG4	1.431	HAP4	1.499	ANX4	1.635
DEJ1	1.326	HAP5	1.680	ANX5	1.703
DEJ2	1.205	HAP6	2.498	ANX6	1.431
DEJ3	1.134	HAP7	1.546	ANX7	1.596
DEJ4	1.834			ANX8	1.368
DEJ5	1.697				

**Table 4:** Reliability and convergent validity: comparison of the initial and final structural model.

Construct	Number of Items		Cronbach’s Alpha		Average Variance Extracted (AVE)		Composite Reliability	
	Initial Model	Final Model	Initial Model	Final Model	Initial Model	Final Model	Initial Model	Final Model
ANX	8	5	0.832	0.847	0.460	0.539	0.872	0.894
HAP	7	7	0.853	0.853	0.537	0.537	0.889	0.889
DEJ	5	4	0.722	0.732	0.482	0.559	0.819	0.833
ANG	4	4	0.763	0.763	0.585	0.585	0.849	0.849

**Table 5:** Discriminant validity: Fornell–Larcker criterion.

Construct	ANG	ANX	DEJ	HAP
ANG	0.765	-	-	-
ANX	0.710	0.734	-	-
DEJ	0.677	0.718	0.748	-
HAP	−0.478	−0.480	−0.421	0.733

**Table 6:** Discriminant validity: heterotrait-monotrait (HTMT) criterion.

Construct	ANG	ANX	DEJ	HAP
ANG	-	-	-	-
ANX	0.898	-	-	-
DEJ	0.897	0.890	-	-
HAP	0.581	0.570	0.504	-

**Table 7:** Hypothesis testing results.

Relationship	Path Coefficient (*β*)	*t*-Statistics	*p*-Value	Test Result: Hypothesis
DEJ → HAP	−0.065	0.822	0.411	H1: unsupported
ANG → HAP	−0.252	3.218	0.001	H2: supported
ANX → HAP	−0.255	3.451	0.001	H3: supported
DEJ → ANX	0.435	7.465	0.000	H4: supported
ANG → ANX	0.416	8.256	0.000	H5: supported
DEJ → ANX→ HAP	−0.111	2.864	0.004	H6: supported
ANG → ANX→ HAP	−0.106	3.394	0.001	H7: supported

## Appendix A

**Table A1:** Model constructs and their corresponding item measures [9].

Construct and Item Measure Description	
**Construct 1: Anxiety (ANX)**	
**ANX1**	“I feel fear at work.”
*** ANX2**	“I feel that matters related to work are getting out of control, which makes me panic.”
**ANX3**	“What is happening at work fills me with anxiety and makes me feel threatened.”
**ANX4**	“I’m thinking that on Monday I need to go to work; I feel anxious.”
**ANX5**	“I have symptoms of anxiety and nervousness at work, and I’m not able to calm down.”
*** ANX6**	“Actions taken by my co-workers and/or supervisors make me feel uncertain.”
**ANX7**	“I am concerned that I won’t be able to meet the work requirements.”
*** ANX8**	“I feel uncertain at work.”
**Construct 2: Happiness (HAP)**	
**HAP1**	“I find my work enjoyable.”
**HAP2**	“My job brings me satisfaction.”
**HAP3**	“My job gives me a sense of fulfilment.”
**HAP4**	“I find contentment in my work.”
**HAP5**	“Overall, I feel relaxed and free.”
**HAP6**	“I am happy with my relations with my superiors.”
**HAP7**	“I have a positive attitude toward the task and problems that I am facing at work.”
**Construct 3: Dejection (DEJ)**	
**DEJ1**	“At work, I feel like I reached the bottom.”
**DEJ2**	“When it comes to my job, it cannot be worse.”
*** DEJ3**	“Most work-related activities make me feel sad and useless.”
**DEJ4**	“I don’t see any career path in front of me.”
**DEJ5**	“I have a sense of being suspended from what is happening at work.”
**Construct 4: Anger (ANG)**	
**ANG1**	“Recently, everything related to my work makes me angry.”
**ANG2**	“I find everything at work annoying.”
**ANG3**	“The tasks I am getting from my supervisor make me furious.”
**ANG4**	“There are moments when I feel very irritated.”

* Items were deleted after the final confirmatory factor analysis.

## References

[1] De Neve J.E., Ward G., Helliwell J., Layard R., Sachs J. (2017). Happiness at Work. World Happiness Report 2017.

[2] Kemper T. (1987). How many emotions are there? Wedding the social and the automatic components. Am. J. Sociol..

[3] Kemper T.D., Stets J.E., Turner J.H. (2006). Power and status and the power-status theory of emotions. Handbook of the Sociology of Emotions. Handbooks of Sociology and Social Research.

[4] Plutchik R. (2001). The nature of emotions. Am. Sci..

[5] Oatley K., Johnson-Laird P.N. (2014). Cognitive approaches to emotions. Trends Cogn. Sci..

[6] Lindebaum D., Jordan P.J. (2014). When it can be good to feel bad and bad to feel good: Exploring asymmetries in workplace emotional outcomes. Hum. Relat..

[7] Lindebaum D., Jordan P.J. (2012). Positive emotions, negative emotions, or utility of discrete emotions?. J. Org. Behav..

[8] Frost P.J. (2003). Toxic Emotions at Work.

[9] Jaworek M.A., Marek T., Karwowski W. (2020). The scale of Work-Related Affective Feelings (WORAF). Appl. Ergon..

[10] Antonacopoulou E.P., Gabriel Y. (2001). Emotion, learning and organizational change: Towards an integration of psychoanalytic and other perspectives. J. Organ. Chang. Manag..

[11] Gooty J., Connelly S., Griffith J., Gupta A. (2010). Leadership, affect and emotions: A state of the science review. Leadersh. Q..

[12] Riforgiate S.E., Komarova M. (2017). Emotion and work. The International Encyclopedia of Organizational Communication.

[13] Ekman P. (2016). What scientists who study emotion agree about. Perspect. Psychol. Sci..

[14] Parkinson B., Fischer A.H., Manstead A.R. (2005). Emotion in Social Relations: Cultural, Group, and Interpersonal Processes.

[15] Hareli S., Rafaeli A., Parkinson B., Ashkanasy N.M., Cooper C.L. (2008). Emotions as social entities: Interpersonal functions and effects of emotion in organizations. Research Companion to Emotion in Organizations.

[16] Zembylas M. (2007). Theory and methodology in researching emotions in education. Int. J. Res. Method Educ..

[17] Russell J.A. (2003). Core affect and the psychological construction of emotion. Psychol. Rev..

[18] Salovey P., Mayer J. (1990). Emotional intelligence. Imagin. Cogn. Personal..

[19] Goleman D. (1995). Emotional Intelligence.

[20] Hochschild A.R. (1983). The Managed Heart.

[21] Sutton R.I., Rafaeli A. (1988). Untangling the relationship between displayed emotions and organizational sales: The case of convenience stores. Acad. Manag. J..

[22] Grandey A.A. (2003). When “the show must go on”: Surface acting and deep acting as determinants of emotional exhaustion and peer-rated service delivery. Acad. Manag. J..

[23] Morris J.A., Feldman D.C. (1997). Managing emotions in the workplace. J. Manag. Issues.

[24] Hochschild A.R. (1979). Emotion work, feeling rules, and social structure. Am. J. Sociol..

[25] Fisher C.D. (2010). Happiness at Work. Int. J. Manag. Rev..

[26] Sargeant J., Mann K., Sinclair D., Van der Vleuten C., Metsemakers J. (2008). Understanding the influence of emotions and reflection upon multi-source feedback acceptance and use. Adv. Health Sci. Educ..

[27] Lazarus R., Cohen-Charash Y., Payne R.L., Cooper C.L. (2001). Descrete emotions in organizational life. Emotions at Work. Theory, Research and Applications for Management.

[28] Izard C.E., Ackerman B.P., Lewis M., Haviland-Jones J.M. (2000). Motivational, organizational, and regulatory functions of discrete emotions. Handbook of Emotions.

[29] Barr-Zisowitz C., Lewis M., Haviland-Jones J.M. (2000). “Sadness”— Is there such a thing?. Handbook of Emotions.

[30] Garson G.D. (2006). Partial Least Squares: Regression and Structural Equation Models.

[31] Hair J.F., Hult G.T.M., Ringle C., Sarstedt M. (2016). A Primer on Partial Least Squares Structural Equation Modeling (PLS-SEM).

[32] Daoud J.I. (2017). Multicollinearity and regression analysis. J. Phys. Conf. Ser..

[33] Fornell C., Larcker D.F. (1981). Evaluating structural equation models with unobservable variables and measurement error. J. Mark. Res..

[34] Cronbach L.J. (1951). Coefficient Alpha and the Internal Structure of Tests. Psychometrika.

[35] Carmines E.G., Zeller R.A. (1979). Reliability and Validity Assessment.

[36] Ringle C.M., Wende S., Becker J.M. (2015). “SmartPLS 3”. www.smartpls.com.

[37] Hair J.F., Sarstedt M., Hopkins L., Kuppelwieser V.G. (2014). Partial least squares structural equation modeling (PLS-SEM). Eur. Bus. Rev..

[38] Henseler J., Ringle C.M., Sarstedt M. (2015). A new criterion for assessing discriminant validity in variance-based structural equation modeling. J. Acad. Mark. Sci..

[39] Russell J.A. (1980). A circumplex model of affect. J. Personal. Soc. Psychol..

[40] Van Katwyk P.T., Fox S., Spector P.E., Kelloway E.K. (2000). Using the job-related affective well-being scale (JAWS) to investigate affective responses to work stressors. J. Occup. Health Psychol..

[41] Basińska B.A., Gruszczyńska E., Schaufeli W.B. (2014). Psychometric properties of the Polish version of the job-related affective well-being scale. Int. J. Occup. Med. Environ. Health.

[42] Fischer A.H., Rodriguez Mosquera P.M., Van Vianen A.E., Manstead A.S. (2004). Gender and culture differences in emotion. Emotion.

[43] Ashkanasy N.M., Daus C.S. (2002). Emotion in the workplace: The new challenge for managers. Acad. Manag. Perspect..

[44] Ashkanasy N.M., Dorris A.D. (2017). Emotions in the workplace. Annu. Rev. Organ. Psychol. Organ. Behav..

[45] Grandey A.A. (2000). Emotional regulation in the workplace: A new way to conceptualize emotional labor. J. Occup. Health Psychol..

[46] Davidson R.J., Pizzagalli D., Nitschke J.B., Putnam K. (2002). Depression: Perspectives from affective neuroscience. Annu. Rev. Psychol..

[47] Davidson R.J., Putnam K.M., Larson C.L. (2000). Dysfunction in the neural circuitry of emotion regulation—A possible prelude to violence. Science.

[48] Eisenberg N. (2000). Emotion, regulation, and moral development. Annu. Rev. Psychol..

[49] Dodge K.A. (1989). Coordinating responses to aversive stimuli: Introduction to a special section in the development of emotion regulation. Dev. Psychol..

[50] Oktug Z. (2013). Managing emotions in the workplace: It is mediating effect on the relationship between organizational trust and occupational stress. Int. Bus. Res..

[51] Rosete D., Ciarrochi J. (2005). Emotional intelligence and its relationship to workplace performance outcomes of leadership effectiveness. Leadersh. Organ. Dev. J..

[52] Zhang L., Yao Y., Yiu T.W. (2020). Job Burnout of Construction Project Managers: Exploring the Consequences of Regulating Emotions in Workplace. J. Constr. Eng. Manag..

